# Gingival Crevicular Blood Glucose as a Novel Method for Screening Diabetes Mellitus in Periodontally Compromised Patients

**DOI:** 10.7759/cureus.39444

**Published:** 2023-05-24

**Authors:** Chandni Patel, Bela Dave, Romil Patel, Santosh Kumar, Vidhi Dattani, Surabhi Joshi, Mainul Haque

**Affiliations:** 1 Department of Periodontology, Karnavati School of Dentistry, Karnavati University, Gandhinagar, IND; 2 Department of Periodontology, Ahmedabad Municipal Corporation Dental College and Hospital, Ahmedabad, IND; 3 Department of Ophthalmology, Dr. M.K. Shah Medical College and Research Centre, Ahmedabad, IND; 4 Department Pharmacology and Therapeutics, National Defence University of Malaysia, Kuala Lumpur, MYS

**Keywords:** minimally invasive, screening method, finger capillary blood glucose, diagnostic tool, glucose monitoring devices, fasting blood glucose

## Abstract

Introduction: Patients with periodontitis are significantly more likely to have undetected diabetes mellitus (DM). Self‑monitoring devices like glucometers provide a simple method for rapid monitoring of the glucose level in blood by utilizing a blood sample from the finger, but this method requires puncturing to obtain blood. Bleeding from the gingival sulcus, obtained during oral hygiene examination, can be utilized for screening DM patients. Therefore, this study was performed with the aim of determining the efficacy of gingival crevicular blood as a non-invasive screening method for DM patients, as well as correlating and comparing gingival crevicular blood glucose (GCBG) levels with finger capillary blood glucose (FCBG) and fasting blood glucose (FBG) among non-diabetic and diabetic group patients.

Methods: In this cross-sectional comparative study, a total of 120 participants having moderate to severe gingivitis/periodontitis with an age range of 40 to 65 years were divided into two groups on the basis of FBG range taken from an antecubital vein: non-diabetic (≤126, n=60) and diabetic (≥126, n=60) groups. Blood oozing during the routine periodontal examination from the periodontal pocket was recorded using a test strip of a glucose self-monitoring device (AccuSure^®^Simple) as GCBG. Concomitantly FCBG was collected from the fingertip. These three parameters were statistically analyzed using the Student's t-test and the one-way ANOVA test and correlated with Pearson's correlation coefficient for both groups.

Results: The mean and standard deviation for the three parameters GCBG, FBG, and FCBG were 93.78±12.03, 89.98±13.22, and 93.08±15.56, respectively, for the non-diabetic group and 154.52±45.05, 159±47.00, and 162.23±50.60 subsequently for the diabetic group. Comparing glucose level parameters among the non-diabetic and diabetic groups suggests a significant difference with the p-value <0.001*(inter-group). ANOVA test was done for both groups suggesting no significant difference among these three methods of measuring blood glucose level, where the p-value found was 0.272 for the non-diabetic and 0.665 for the diabetic group (intra-group comparison). Pearson's correlation values suggested a good positive correlation for the non-diabetic group, with parameters GCBG and FBG (r=0.864), GCBG and FCBG (r=0.936), and FBG and FCBG (r=0.837). The diabetic group's Pearson's correlation suggested a highly significant positive correlation between three different methods in which GCBG and FBG (r=0.978), GCBG and FBG (r=0.977), and FBG and FCBG (r= 0.982).

Conclusion: Blood oozing from the periodontal pocket during routine oral hygiene examination can be utilized by dental healthcare professionals to screen pre-diabetic patients which can be used as a simple and less invasive method for DM patients.

## Introduction

Diabetes mellitus (DM) is one of the most common chronic health conditions in today’s civilization, imparting significant pressure and burdens on any healthcare system worldwide. DM is characterized by continuously high glucose levels associated with variations in the metabolism of carbohydrates, fats, and proteins. DM is heterogeneous in nature, with abnormal insulin secretion, which leads to hyperglycemia and may cause damage to organs such as the heart, eyes, nerves, arteries, and kidneys. Furthermore, these organs are vulnerable to organ failure in chronic DM [1].

As lifestyles have changed, such as increased physical inactivity and sleep disturbances, the prevalence of DM among the adult population between 18 years and older has reached 10.5% [2]. Various factors are responsible for increasing cases of DM, such as altered food habits, stress, and obesity. By 2030, the number of cases of DM is estimated to reach 438 million, and, at present, DM is the sixth leading cause of death among older patients [3].

DM patients are associated with a plethora of oral manifestations, such as burning mouth syndrome, xerostomia, increased number of dental caries, destructive periodontitis, alteration in taste, and fungal infections as routinely observed by oral healthcare professionals [3]. DM screening is often made by perceiving oral manifestations, signs, and symptoms as reported by patients and blood analyses [4]. In 2021, the American Diabetes Association (ADA) gave updated diagnostic guidelines for DM, aiming to assist in differentiating between pre-diabetes and diabetes patients [5].

The traditional laboratory techniques used to measure venous blood glucose or hemoglobin A1C (HbA1c) or glycated hemoglobin regarding DM assessment takes time and require sophisticated equipment. Small, portable glucometers have made it possible for diabetics to have considerably more control over their condition. Glucometers measure blood glucose levels in a matter of seconds using a small drop of capillary blood from a finger stick sample and it equals the venous blood glucose measurement [6].

As the prevalence of DM is increasing worldwide, early detection has become crucial. Moreover, patients usually remain asymptomatic during the early stages of DM [7]. Blood from the gingival sulcus, which is a mixture of capillary blood and gingival crevicular fluid (i.e., an inflammatory exudate), can be used for painless monitoring of blood glucose levels with glucometers for suspected patients [8]. Thus, blood collected from deep periodontal pockets typically bleeds while probing for routine periodontal examination by oral healthcare professionals can be used for screening DM patients, as compared to more invasive methods such as finger-puncture with a sharp lancet or laboratory investigation [9].

In this study, we aimed to evaluate the efficacy of using gingival crevicular blood for the determination of blood glucose levels compared with other, more invasive, methods.

## Materials and methods

Study population

This cross-sectional comparative study was conducted at the Karnavati School of Dentistry’s Department of Periodontology in Gandhinagar, Gujarat, India, with approval from the institution’s ethical council (KSDEC/22-23/Apr/012). The study population included 120 participants aged 40-65 years who visited the Department of Periodontology, Gandhinagar, from December 2021 to December 2022. All participants provided their written, informed consent at the outset of the study. The participants provided verbal responses to a questionnaire about their brief medical, previous dental, and family histories. These data were collected and recorded into a proforma along with each participant’s name, age, gender, and pertinent medical history. Pre-informed consent was obtained from patients and their relatives for the data to be published.

Study groups

**Figure 1:**
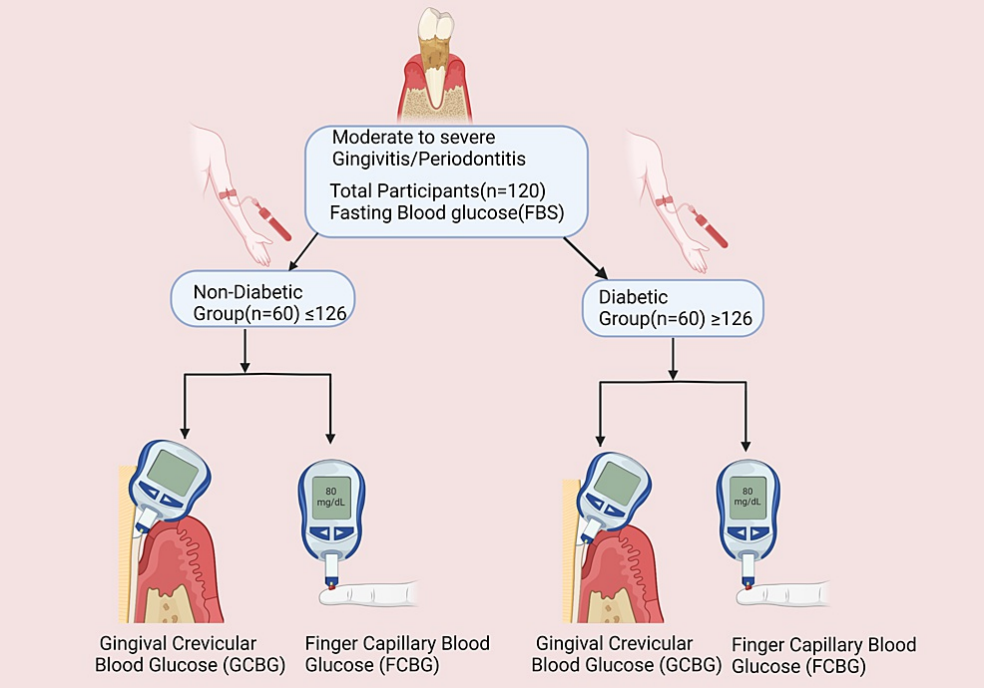
Flow chart illustrating the division of gingivitis/periodontitis patients into non-diabetic and diabetic groups on the basis of FBG. GCBG and FCBG measurements are then taken for both groups simultaneously. This figure has been drawn utilizing the premium version of Bio Render with license number WW25CNSTQO Image credit: Dr. Chandni Patel

Participants with moderate to severe gingivitis/periodontitis diagnosed according to the World Workshop on Periodontology classification (2017) [10] and fulfilling the ADA [5] criteria who were willing to take part were enrolled in the study (Figure 1). The participants were told not to take food for at least eight hours prior to giving blood samples for laboratory investigation. As shown in the flow chart found in Figure 1, group categorization was done based on fasting blood glucose (FBG) level measurement: with ≥126mg/dL indicating the diabetic group and ≤126mg/dL indicating the non-diabetic group. Then, gingival crevicular blood glucose (GCBG) samples from the gingival sulcus and finger capillary blood glucose (FCBG) samples from the fingertip were collected and tested using an AccuSure® Simple glucometer for both groups.

Participants suffering from any other systemic diseases other than DM were excluded from the study. Patients taking medications such as anticoagulant therapy, salicylates, acetaminophen, ascorbic acid, or other sugar-lowering agents, in addition to oral anti-diabetic agents, were excluded from the study. Furthermore, individuals who chewed tobacco, smoked, or frequently consumed alcohol were excluded from the study.

Clinical and laboratory investigations

Fasting Bood Glucose Measurement

Patients with moderate to severe gingivitis/periodontitis were examined for clinical parameters such as bleeding on probing. Then, the patients were divided into groups based on FBG measurements. Preferably, the non-dominant arm’s ante-cubital fossa was used to collect blood samples for intra-venous blood investigations. FBS values for all patients in the diabetic and non-diabetic groups were noted and tabulated.

GCBG Measurement

Patients were then informed to rinse their mouths with 0.2% chlorhexidine mouthwash to prevent contamination. GCBG examination was done using the UNC-15 probe by Qulix™ from the Hu-Friedy group. Upper front segment teeth showing blood oozing from the sulcus were usually preferred. The selected sites were then isolated with cotton rolls. The AccuSure® simple glucometer was directly placed into the sulcus showing an oozing blood site, and glucose measurements were recorded in units of mg/dL from the glucometer screen after the device beeped.

Blood Glucose Assessment (Fingertip Capillary Blood)

Then, FCBG levels were measured at the patients' fingertips. The research participants were distinctly explained about the method of fingertips blood glucose assessment. The finger capillary blood was taken from the non-dominant hand. All patients’ middle fingertips were rubbed with cotton using a surgical spirit and avoid the first blood drop to improve the precision of the findings. The AccuSure® simple glucometer was kept ready as soon as another drop from the fingertip came, and the test strip was held at the site until the device beeped and the value was seen on the screen, which was recorded as FCBG in units of mg/dL.

Statistical Methods

Each clinical parameter’s mean and standard deviation were computed. The values were compiled, and the means and standard deviations of glucose measurements of FBG, GCBG, and FCBG were calculated for both groups. For comparative evaluation, the FBG, GCBG, and FCBG parameters of the diabetic and non-diabetic groups were assessed with Student’s t-test (inter-group). One-way ANOVA was done to compare the results of the three methods among both groups (intra-group). Pearson’s correlation coefficient (r) was used to determine any correlation between the three techniques.

## Results

A total of 120 patients who fulfilled the criteria were enrolled in the study, comprising 68 women and 52 men. The means and standard deviations (mean±SD) for FBG, GCBG, and FCBG levels were 89.98±13.22, 93.78±12.03, and 93.08±15.56 mg/dL for the non-diabetic group and 159.68±47.00, 154.52±45.05, and 162.23±50.60 mg/dL for the diabetic group, respectively (Table1).

**Table 1:** Student’s t-test for non-diabetic and diabetic groups (inter-group comparison) FBG: fasting blood glucose; GCBG: gingival crevicular blood glucose; FCBG: finger capillary blood glucose

Parameter	Group	n	Mean (mg/dL)	Std. deviation (mg/dL)	Std. error mean (mg/dL)	p-value
FBG	Non-diabetic	60	89.9833	13.2262	1.707	0.001*
	Diabetic	60	159.68	47.010	6.069	
GCBG	Non-diabetic	60	93.7833	12.0396	1.554	0.001*
	Diabetic	60	154.5277	45.058	5.81700	
FCBG	Non-diabetic	60	93.0833	15.5676	2.009	0.001*
	Diabetic	60	162.23	50.606	6.533	

Student’s t-test was performed between the non-diabetic and diabetic groups, where differences in parameters were considered statistically significant when p≤0.001. The one-way ANOVA used to assess differences between the three different glucose-measuring methods within groups showed p-values of 0.27 for the non-diabetic group and 0.66 for the diabetic group, suggesting non-significant differences between the three methods (Table 2).

**Table 2:** ANOVA test for blood glucose levels among non-diabetic and diabetic groups (intra-group comparison) FBG: fasting blood glucose; GCBG: gingival crevicular blood glucose; FCBG: finger capillary blood glucose

Group	Parameter			p-value
	GCBG	FBG	FCBG	
Non-diabetic	93.78±12.03	89.98±13.22	93.08±15.56	0.2725 (NS)
Diabetic	154.52±45.05	159.68±47.01	162.23±50.60	0.6657 (NS)

Pearson’s correlation coefficients were determined for the three glucose screening techniques used to assess the blood glucose levels of the DM patients. In the non-diabetic group, when comparing the GCBG values with those of the other two methods, positive correlations were found between GCBG and FBG (r=0.864) and GCBG and FCBG (r=0.936). We also found an r-value of 0.837 when comparing FBG with FCBG, suggesting a positive correlation (Table 3).

**Table 3:** Pearson’s correlation coefficients for the non-diabetic group FBG: fasting blood glucose; GCBG: gingival crevicular blood glucose; FCBG: finger capillary blood glucose. **p-values are significant at <0.01

Parameter	Pearson’s correlation coefficient	FBG	FCBG
GCBG	R	0.864	0.936
	P	0.0000**	0.0000**
FBG	R	-	0.837
	P	-	0.0000**

Compared with the non-diabetic group, the diabetic group showed a highly significant correlation between FBG, GCBG, and FCBG, with similar r-values among the different methods used (Table 4). The r-values were 0.978, 0.977, and 0.982 for GCBG and FBG, GCBG and FBG, and FBG and FCBG, respectively. Pearson’s correlation coefficient was significant among the three methods at p<0.01. Even with increased sugar levels in both groups, the three methods showed no significant difference in measuring glucose levels.

**Table 4:** Pearson’s correlation coefficients for the diabetic group FBG: fasting blood glucose; GCBG: gingival crevicular blood glucose; FCBG: finger capillary blood glucose. **p-values are significant at <0.01

Parameter	Pearson’s correlation coefficient	FBG	FCBG
GCBG	R	0.978	0.977
	P	0.0000**	0.0000**
FBG	R	-	0.982
	P	-	0.0000**

## Discussion

Various laboratory investigation methods are available for the diagnosis of DM. Invasive methods for measuring blood sugar levels include determining random, fasting, postprandial glucose levels, or HbA1c. These methods increase patients' suffering and discomfort both physically and mentally. Oral healthcare professionals can play a vital role in screening DM by using less invasive techniques. Periodontitis, the sixth most common complication of DM, is routinely seen in DM patients [11]; such patients produce a good amount of blood due to inflammation in the periodontium, which can be utilized for screening [12]. Patients with diabetes are routinely treated by a periodontist using incomplete data on blood glucose management [13]. Kassim et al., in their cross-sectional study, suggested that the majority of dentists are willing to incorporate medical screening in their practice but lack awareness about screening methods [14]. Although we used a glucometer in our study, compared with routine laboratory investigation methods, these self-glucose-monitoring devices typically have an error of only 15% or less, as specified by the ADA [15].

Measurements of crevicular blood glucose levels have been made in the past and have exhibited agreement with finger-stick blood glucose levels, indicating that taking blood from the gingival sulcus or crevice testing may be a useful technique in identifying pre-diabetic or borderline-diabetic patients [16]. A study by Masiero et al. suggested that diabetes screening made it possible for a predictable percentage of individuals to become aware of their pathological or pre-pathological condition and seek appropriate and prompt medical care [17]. In their systematic review and meta-analysis, Yonel et al. suggested that utilizing dental professionals to find cases of undiscovered type 2 DM may be advantageous [18].

In our study, we divided the patients into two groups, which made it easy to record data and determine any variation in parameters between groups. Means and standard deviations of blood glucose values were used in Student’s t-tests between both groups. We found higher sugar values for finger capillary blood compared with that of gingival crevices, which might be due to saliva or plaque contamination. To prevent this effect, we instructed the patients to use mouth rinse prior to blood collection. Similar to our study, an ANOVA test done by Partheeban et al. also found p-values of 0.77 between GCBG and VCBG in non-diabetic patients and 0.96 between GCBG and FCBG in diabetic patients [19]. Among the 60 non-diabetic patients, there were positive correlations between FCBG and VCBG (r=0.837), VCBG and GCBG (r=0.864), and GCBG and FCBG (r=0.936), which is similar to previous studies by Patil et al. [20], Parihar et al. [21], and Grigoriadis et al. [22]. The diabetic group showed stronger correlations between GCBG and VCBG (r=0.978) and GCBG and FCBG (r=0.977), similar to recent studies by Vummidi et al. [23] (r=0.924) and Sibly et al. [24] (r=0.97), respectively.

Earlier studies on measuring sulcular blood glucose suggested that it is correlated with finger-stick glucose, but we also tested venous blood, as it is considered the gold standard. It was critical that samples were collected in sterile and isolated premises, as the presence of saliva or plaque will affect the results. Therefore, we attempted to maintain proper isolation by using cotton rolls and suction. As patients are often afraid and nervous during any surgical procedure due to their previous experience, we attempted to use less invasive methods. We did not include Hba1c in this study, even though it is considered more accurate and estimates average blood glucose at a low cost.

Routine dental check-ups by dentists and dental hygienist teams can play a role in knowing blood sugar levels and assist in DM screening by referring patients to physicians. By using the technique given in this study, we were able to easily measure blood glucose levels from gums in a relatively less invasive way. Surgical procedures such as periodontal flap surgery and implant placement procedures for uncontrolled diabetic patients can also be performed by dentists by easily determining blood sugar levels in dental offices with safe chair-side measurements.

Limitations

This study was limited in a few ways, including its small sample size. A study involving a larger number of participating patients should be done to confirm the reliability of GCBG. In addition, the laboratory analysis was done in the fasting stage, which sometimes requires a higher number of visits for the patients. Finally, postprandial laboratory investigations can be done in future studies to determine whether food and patient blood sugar levels are correlated with each other.

## Conclusions

As per the findings of the current investigation, diabetic patients can be screened using GCBG expression during normal periodontal examination. A chair-side screening at dental offices during routine oral health examination is potentially more helpful compared to more time-consuming, intrusive procedures, and it offers a more objective indicator for a physician referral. Consequently, by taking part in the search for undiagnosed asymptomatic DM, the dentist may become an even more vital component of the healthcare team.
